# Growth of PIK3CA-driven cerebral cavernous malformations does not require microbiome stimulation

**DOI:** 10.1172/jci.insight.208733

**Published:** 2026-07-16

**Authors:** Lun Li, Rhonda Lightle, Bader Ali, Georgeio Sader, Robert Shenkar, Sean P. Polster, Douglas A. Marchuk, Jan-Karl Burkhardt, Issam A. Awad, Mark L. Kahn

**Affiliations:** 1 Cardiovascular Institute and Department of Medicine, Perelman School of Medicine, and; 2 Department of Neurosurgery, Perelman School of Medicine, Hospital of the University of Pennsylvania, University of Pennsylvania, Philadelphia, Pennsylvania, USA.; 3 Department of Neurological Surgery, Section of Neurovascular Surgery, The University of Chicago Medicine and Biological Sciences, Chicago, Illinois, USA.; 4 Department of Molecular Genetics and Microbiology, School of Medicine, Duke University, Durham, North Carolina, USA.

**Keywords:** Neuroscience, Vascular biology, Microbiome, Stroke

## Abstract

We find that the microbiome plays a critical role in the formation of cerebral cavernous malformations in neonatal but not adult life.

Cerebral cavernous malformations (CCMs) are vascular lesions causing intracerebral hemorrhage, seizures, and progressive neurological deficits (1). Human genetic studies show that CCMs arise following endothelial loss-of-function (LOF) mutations in *KRIT1*, *CCM2*, or *PDCD10*, or an I441M gain-of-function (GOF) mutation in *MAP3K3*, resulting in augmented MEKK3/KLF2/4 signaling (1). CCMs present as a familial form with germline mutations conferring multiple lifelong lesions, or a sporadic form with single lesions from acquired mutations. Recently, clinically symptomatic CCMs were found to harbor oncogenic *PIK3CA* GOF mutations (2).

Mouse models demonstrated that neonatally induced endothelial loss of *KRIT1*, *CCM2*, or *PDCD10* is sufficient to confer widespread CCMs that histologically resemble human lesions. While these models yielded key mechanistic insights, they diverge from human sporadic CCMs because CCM gene loss is induced only during early postnatal life and not in the mature brain. This was later explained by the discovery that more than 70% of surgically resected CCMs harbor *PIK3CA* mutations and by studies demonstrating that combined CCM LOF and PI3K GOF drive rapid solitary lesion growth in the mature mouse brain (2).

In neonatally induced mouse models, CCM formation unexpectedly requires brain endothelial TLR4 signaling triggered by circulating gut microbiome–derived lipopolysaccharide (LPS) (3). Our group demonstrated that neonatally induced CCM formation depends on both brain endothelial TLR4 and the microbiome, while lesion burden in human familial CCMs correlates with genomic changes enhancing TLR4 signaling (3). These findings were substantiated by the finding that germline mutations in *PDCD10* confer more severe disease than *KRIT1* or *CCM2*, as *PDCD10* also maintains gut barrier integrity, limiting microbial dissemination and circulating LPS (4). The discovery of a gut-brain signaling axis raised the possibility that CCM risk is determined by — and could be modified by — an individual’s microbiome.

The discovery that many resected human CCMs harbor a second *PIK3CA* GOF hit, and that lesions form only during neonatal life without such a hit, raised the possibility that microbiome-driven CCM formation is restricted to neonatal life. To test this, we used an inducible adult CCM model in which brain endothelial *Krit1* LOF and *Pik3ca*^^H1047R^^ GOF are induced focally by AAV-Cre injection following craniotomy. To assess microbiome contribution, 8- to 10-week-old *Krit1^fl/fl^*; i*Pik3ca*^^H1047R^^ mice received antimicrobial treatment previously shown to deplete Gram-negative bacteria (GNB) and prevent neonatally induced CCM formation (3) (Figure 1A). Following a 3-day pretreatment, CCM formation was induced by craniotomy and AAV-Cre injection. Fecal bacterial load and GNB abundance, measured by qPCR of total bacterial 16S and Bacteroidetes s24-7, respectively, were reduced by more than 99% before lesion induction (Figure 1, B and C). Lesion growth was monitored longitudinally by live imaging and high-frequency transcranioplasty ultrasound (TCUS), and endpoint lesion volumes quantified by blinded microCT on postoperative day (POD) 21 (Supplemental Figure 1 and Figure 1, D and E; supplemental material available online with this article; https://doi.org/10.1172/jci.insight.208733DS1).

Despite sustained microbiome depletion throughout the 21-day period (Figure 1, B and C), adult i*Pik3ca^H1047R^*-driven CCM lesions formed and progressed at rates indistinguishable from controls (Figure 1, D and E). Live imaging demonstrated variable lesion enlargement over time, with no difference between groups (Supplemental Figure 1 and Figure 1D), and blinded microCT assessment confirmed no significant difference in lesion burden (Figure 1F). The study was powered to detect a large effect on lesion burden, comparable to neonatally induced studies (Cohen’s *d* ≈ 1.3 at 80% power). These findings suggested that, unlike neonatally induced CCM formation due to CCM LOF alone, microbiome-derived LPS is dispensable for adult CCM formation driven by combined CCM LOF and PI3K GOF.

To further test whether the microbiome-derived LPS contributes to i*Pik3ca^H1047R^*-driven CCM formation in adults, we examined the requirement for TLR4, a receptor required in a dose-dependent manner for neonatally induced CCM formation (3). Littermate mice harboring *Krit1^fl/fl^* and *Pik3ca*^H1047R^ alleles on the *Tlr4*^+/+^, *Tlr4*^+/–^, or *Tlr4*^–/–^ background were analyzed as described above. Neither partial nor complete TLR4 loss reduced lesion burden or delayed lesion onset (Supplemental Figure 2), supporting the notion that neither the microbiome nor LPS/TLR4 signaling is required for CCM formation in the adult model.

Although these are negative findings, they provide valuable insights into CCM pathogenesis, therapeutic strategies, and the use/limitations of mouse models. The neonatally induced CCM model relies on both active brain angiogenesis that elevates endothelial PI3K signaling and an immature gut that likely elevates circulating LPS to drive endothelial TLR4/MEKK3 signaling (3). Even in familial CCMs, recent human genetic studies suggest that lesions that enlarge and become symptomatic later in life often harbor *PIK3CA* mutations and may grow independently of the microbiome (1). Therapeutically, targeting the microbiome or TLR4 signaling is unlikely to be effective beyond neonatal life, whereas inhibition of the common PI3K/mTOR pathway remains promising. Finally, these studies emphasize the importance of reevaluating disease models in light of emerging data and approaches.

We acknowledge several limitations. Not all resected CCMs harbor detectable *PIK3CA* mutations (2), and patients with CCM exhibit distinct gut microbiota compared with controls (5). Thus, our findings may be limited to *PIK3CA*-driven lesions. Other factors converging on PI3K signaling may promote CCM growth independently of detectable *PIK3CA* mutations. LPS exposure during infection or chronic intestinal disease could also stimulate growth of otherwise quiescent lesions (6). Analyses of *PIK3CA* mutation burden in growing versus stable CCMs and multifocal CCM models combining CCM LOF and *PIK3CA* GOF should clarify these mechanisms.

## Conflict of interest

IAA reports consulting for Neurelis and Ovid Therapeutics, and JKB for Recursion, outside the submitted work.

## Funding support

This work is the result of NIH funding, in whole or in part, and is subject to the NIH Public Access Policy. Through acceptance of this federal funding, the NIH has been given a right to make the work publicly available in PubMed Central.

- NIH grants R01HL094326 and R01NS100949 (to MLK).
- NIH grant P01NS092521 (to MLK, IAA, and DAM).
- Leducq Foundation (to MLK).
- Adelman CCM fund (to JKB).

## Supplementary Material

Supplemental data

Supporting data values

## Figures and Tables

**Figure 1:**
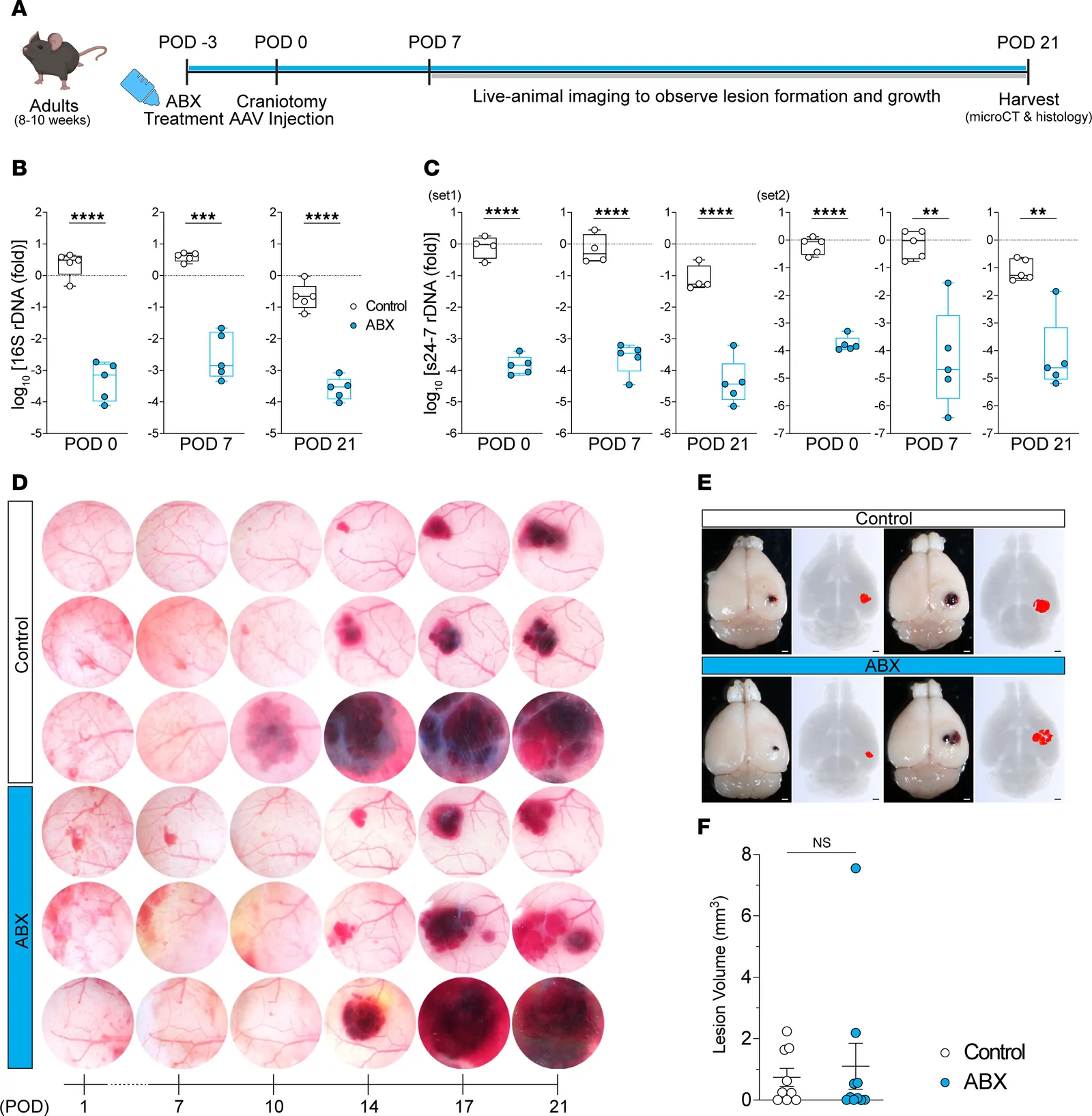
Antibiotic treatment does not prevent *PIK3CA*-driven CCM growth in the adult brain. (**A**) Adult *Krit1^fl/fl^*; i*Pik3ca*^H1047R^ mice received antibiotics (ABX) before craniotomy and focal AAV-Cre injections. (**B** and **C**) qPCR quantification of bacterial 16S rRNA (**B**) and Bacteroidetes s24-7 rRNA (**C**). *n* = 5/group. (**D**) Representative CCM preogression. (**E**) Representative gross and microCT images. (**F**) Lesion volumes on POD 21. Control, *n* = 9; ABX, *n* = 10. **P < 0.01, ***P < 0.01, ****P < 0.0001. NS, no significant difference (*P* = 0.6620).

## Version 1 07/16/2026

In-Press Preview

## Version 2 09/08/2026

Electronic publication
