# Supplementary material for: Growth of PIK3CA-driven cerebral cavernous malformations does not require microbiome stimulation
Source: JCI Insight. 2026 Jul 16;11(17):e208733. doi: 10.1172/jci.insight.208733 (PMC13678643; doi:10.1172/jci.insight.208733)
Supplement: Supplementary file 1 [file jciinsight-11-208733-s014.pdf]

Supplemental Material

Methods

Mice

Krit1$^{fl/fl}$; *R26-LSL-Pik3ca*^{H1047R} (*Krit1*$^{fl/fl}$; i*Pik3ca*^{H1047R}) transgenic mice (2), with or without additional *TLR4*$^{-/-}$ alleles (3), were maintained on a mixed genetic background. Mice were housed in individually ventilated cages in a pathogen-free vivarium on a 12:12h light-dark cycle, with food and water provided *ad libitum*.

Sex as a biological variable

Our study examined both male and female mice, aged 8 to 10 weeks, and similar findings are reported for both sexes.

Craniotomy surgery

Animals were anaesthetized with isoflurane (3.0% induction, 1.0-2.0% maintenance in 1.0 l/min oxygen) for surgery. Mice were placed on a heating pad covered with a cotton pad to maintain a body temperature of 37.5 °C. The depth of anesthesia was periodically monitored by testing the animal's reflexes through toe pinch. Ophthalmic lubricant was applied to the eyes to prevent dryness. A stereotactic frame (Stoeling Instruments) was used to immobilize the head with ear bars. Mice undergoing craniotomies received 1.5 mg/kg of dexamethasone intramuscularly (Sigma) and 5 mg/kg of 10% meloxicam (Midwest Vet Supply) subcutaneously. After immobilization, the scalp hair was removed with Nair, and the scalp was cleaned and sterilized with betadine followed by 70% ethanol, repeated at least three times. The

scalp was then removed to expose the skull. The periosteum was carefully removed with blunt forceps. The outer table of the skull was thinned using a handheld high-speed dental drill (Foredom) using a 0.5 mm burr. For adult mice, a 4 mm diameter circular craniotomy was performed over the right somatosensory cortex posterior to the coronal suture. Sterile saline was used to irrigate the drill burr, preventing bone residue buildup and drill overheating. Gelfoam in sterile saline was applied for hemostasis and to remove bone dust. The border of the craniotomy was thinned until the inner table fractures. The craniotomy border was thinned until the inner table fractured. A fine-tip forceps (Fine Science Tools) or up-angled curette (Fine Science Tools) was used to lift the bone flap and gently separate it from the dura. Gelfoam was used to maintain hemostasis and prevent the brain surface from desiccating.

Following the completion of intraparenchymal viral injection, hemostasis was obtained. A cranial window, made from a 5 mm glass coverslip (Warner), was placed over the craniotomy site and secured to the skull using cyanoacrylate glue and dental cement (Keystone). For ultrasound studies, a cranial window made of polymethyl methacrylate (PMMA, customized and donated by Longeviti Neuro Solutions, LLC, Baltimore, MD) was applied instead of glass coverslip. Immediately following surgery, mice were monitored closely and kept separately from their littermates until they were awake and mobile.

Adeno-associated virus (AAV) injection

Recombinant AAV(BR1)-CAG-Cre (AAV-Cre) was purchased from SignaGen Laboratories (#SL116069). Immediately following craniotomy, recombinant AAV vectors

carrying Cre recombinase under the control of the CAG promoter were injected into the right somatosensory cortex centered on +1.5 mm lateral, −1.0 mm posterior to bregma at a depth of 0.5 mm using a beveled glass micropipette. A total of 1 x 10^9 viral genomes in 100 nl sterile PBS were gradually injected over 5 minutes for each mouse to enhance viral diffusion and minimize damage to brain tissue.

Antibiotic administration

Mice were randomly assigned into two groups and received either antibiotic treatment or vehicle via drinking water mixed with 40 g/L of sucralose and red food coloring. Both antibiotic and vehicle water were replaced daily. The following antibiotics weremixed with 0.22 μm filtered water: penicillin (500 mg/L), neomycin (500 mg/L), streptomycin (500 mg/L), metronidazole (1 g/L) and vancomycin (1 g/L). All antibiotics were purchased from the Hospital of the University of Pennsylvania pharmacy.

Bacterial DNA extraction and bacterial ribosomal DNA qPCR.

Fresh fecal pellets were collected from experimental mice at 4 different time points: POD -3, before antibiotic administration; POD 0, day of surgery and AAV-Cre injection; POD 7, the earliest day could observe visible CCM lesions through the cranial window; and POD 21, before tissue harvest at the terminal endpoint. Collection was performed between 10:00 and 12:00, pellets were immediately snap-frozen on dry ice, and stored at -80 °C. The QIAamp DNA Stool Mini Kit (Qiagen 51504 or 51604) was used to extract bacterial DNA from the fecal pellets. Before commencing the standard Qiagen protocol, the frozen fecal pellets was mixed in the included stool

lysis buffer and homogenized with a 5 mm stainless steel bead in a TissueLyser LT (Qiagen 69980) at 50 Hz for 10 min at 4 °C. Concentration of the extracted DNA was equalized and 100 ng of DNA was used per qPCR reaction with universal bacterial 16S rRNA gene primers, two different sets of previously characterized Bacteroidetes s24-7 primers (3). Universal 16S rRNA forward: 5′-ACTGAGAYACGGYCCA-3′; universal 16S rRNA reverse: 5′-TTACCGCGGCTGCTGGC-3′; Bacteroidetes s24-7 rRNA set 1 forward: 5′-GGAGAGTACCCGGAGAAAAAGC-3′; Bacteroidetes s24-7 rRNA set 1 reverse: 5′ TTCCGCATACTTCTCGCCCA-3′; Bacteroidetes s24-7 rRNA set 2 forward: 5′-CCAGCAGCCGCGGTAATA-3′; Bacteroidetes s24-7 rRNA set 2 reverse: 5′-CGCATTCCGCATACTTCTC-3′.

***In vivo* high-frequency transcranioplasty ultrasound (TCUS) imaging and volumetric measurement**

For all experiments using live animal ultrasound imaging, mice were anesthetized with isoflurane (3.0% induction, 1.0-2.0% maintenance in 1.0 l/min oxygen) and placed on a heating pad covered with a cotton pad to maintain a body temperature of 37.5 °C. Warm acoustic gel was applied to the scan field as couplant. Echo with ultrasounds of frequencies ranging from 50 to 60 MHz was performed using Visual Sonic Vevo 2100 system equipped with MS700 transducer (VisualSonics). Real-time B-mode cine-loops clips with the transducer index mark orientation were recorded. Images were acquired and analyzed using Vevo Lab software (VisualSonics). CCM lesion volume was calculated using the formula $V=4/3 \times \pi \times L/2 \times W/2 \times H/2$.

Contrast-enhanced microcomputed tomography (microCT) and volumetric quantification

For all experiments utilizing microCT quantification of CCM lesion volume, brains were harvested and stored in 4% (*w*/*v*) PFA/PBS (pH 7.4). The brains were kept in fixative until staining with non-destructive iodine contrast, followed by microCT imaging, as previously described (2, 3). Tissue processing, imaging, and volume quantification were performed in a blinded manner by investigators at the University of Chicago, with no knowledge of genotype or experimental details.

Statistics

No statistical methods were used to predetermine sample size. All experimental and control animals were littermates, and none was excluded from analyses at the time of harvest. Data were analyzed using GraphPad Prism software and are presented as means ± SEM. *P* values were calculated using two-tailed unpaired Welch's *t*-test and Welch ANOVA followed by Dunnett's T3 *post-hoc* multiple comparison test. *P* values of less than 0.05 were considered statistically significant and are denoted as follows: $^{**}p < 0.01$, $^{***}p < 0.001$, and $^{****}p < 0.0001$.

Study approval

All animal experiments were performed in accordance with institutional guidelines and were approved by the Institutional Animal Care and Use Committee (IACUC) of the University of Pennsylvania. Mice were housed in a pathogen-free vivarium accredited

by the Association for Assessment and Accreditation of Laboratory Animal Care (AAALAC). This preclinical study conformed to the updated ARRIVE (Animal Research: Reporting of In Vivo Experiments) 2.0 guidelines.

Data and materials availability

Values for all data points in graphs are reported in the Supporting Data Values file. Additional raw data, materials, and protocols are available from the corresponding author upon reasonable request.

Author contributions

LL conceived and designed the study, curated and analyzed data, conducted experiments, developed methodology, administered the project, validated results, prepared visualizations, and wrote the original draft, and reviewed and edited the manuscript. RL, BA, GS, RS, and SP performed formal analysis, conducted experiments, developed methodology, validated results, prepared visualizations, and reviewed and edited the manuscript. DAM contributed to study conception, acquired funding, supervised the research, and reviewed and edited the manuscript. IAA contributed to study conception, acquired funding, performed formal analysis, developed methodology, validated results, supervised the research, and reviewed and edited the manuscript. JKB contributed to study conception, developed methodology, validated results, supervised the research, and reviewed and edited the manuscript. MLK conceived and designed the study, acquired funding, developed methodology, administered the project, provided resources, supervised the research, and wrote the

original draft, and reviewed and edited the manuscript. All authors read and approved the final manuscript.

Acknowledgments

We thank the University of Chicago PaleoCT core facility for expert technical assistance with microCT imaging and quantitative image analysis. Transcranial ultrasound imaging was performed by the Rodent Cardiovascular Phenotyping Core (RRID: SCR_022419) at the University of Pennsylvania, supported by the Penn Cardiovascular Institute and NIH grant S10OD016393.

Supplemental Figures

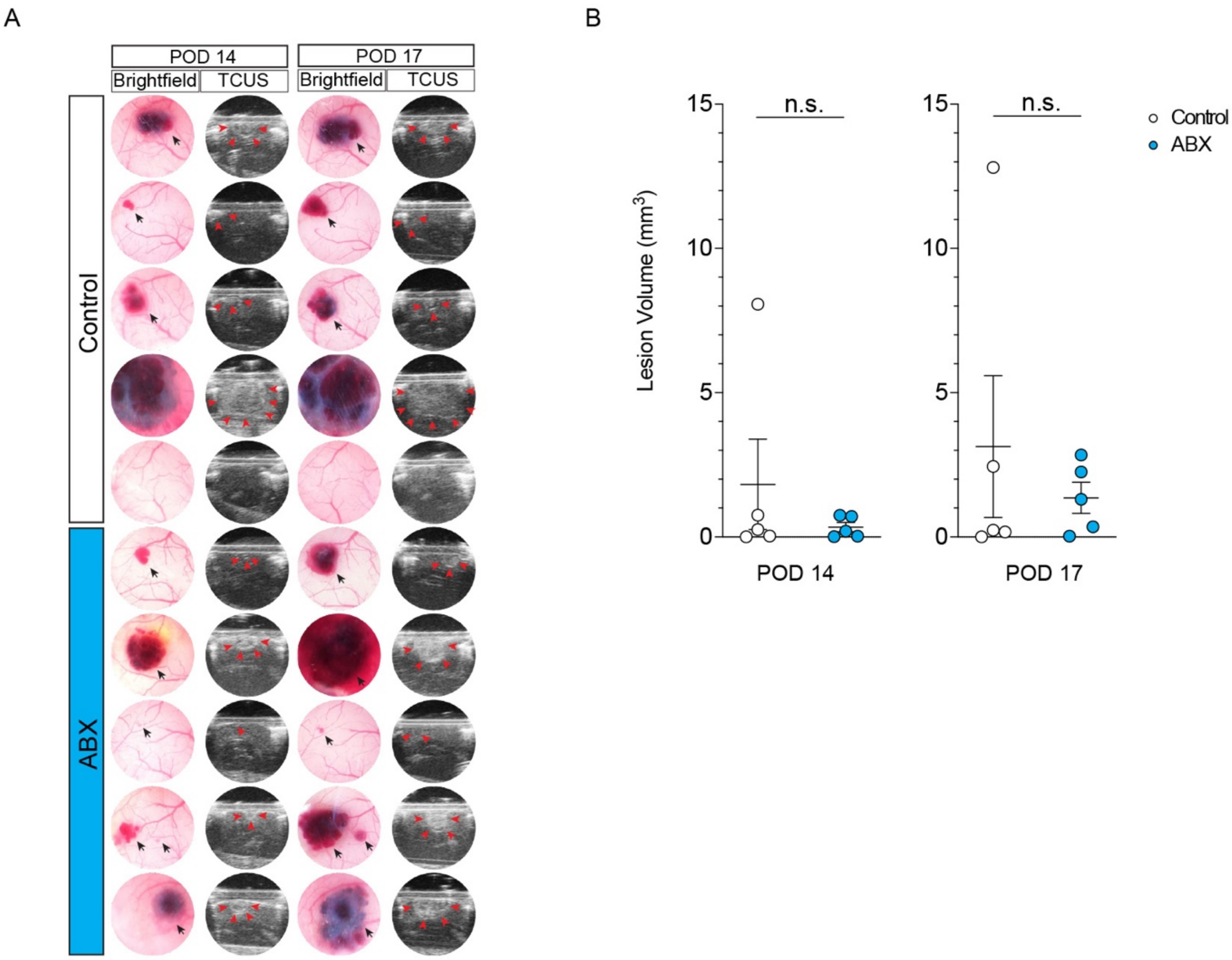

Supplemental Figure 1. Live-animal imaging of CCM growth with ultrasound-based lesion volumetric assessment. (A) Representative microscopic (left) and ultrasonic images through the PMMA cranial windows showing CCM growth in either control or ABX-treated mouse brains on POD 14 and POD 17. (B) Lesion volumes of CCMs were calculated using the formula V = 4/3 × π × L/2 × W/2 × H/2. N=5 per group were analyzed. No statistically significant (n.s.) differences are observed in both data set by unpaired 2-tailed Welch *t* test. Left: p = 0.3988. Right: p = 0.5165.

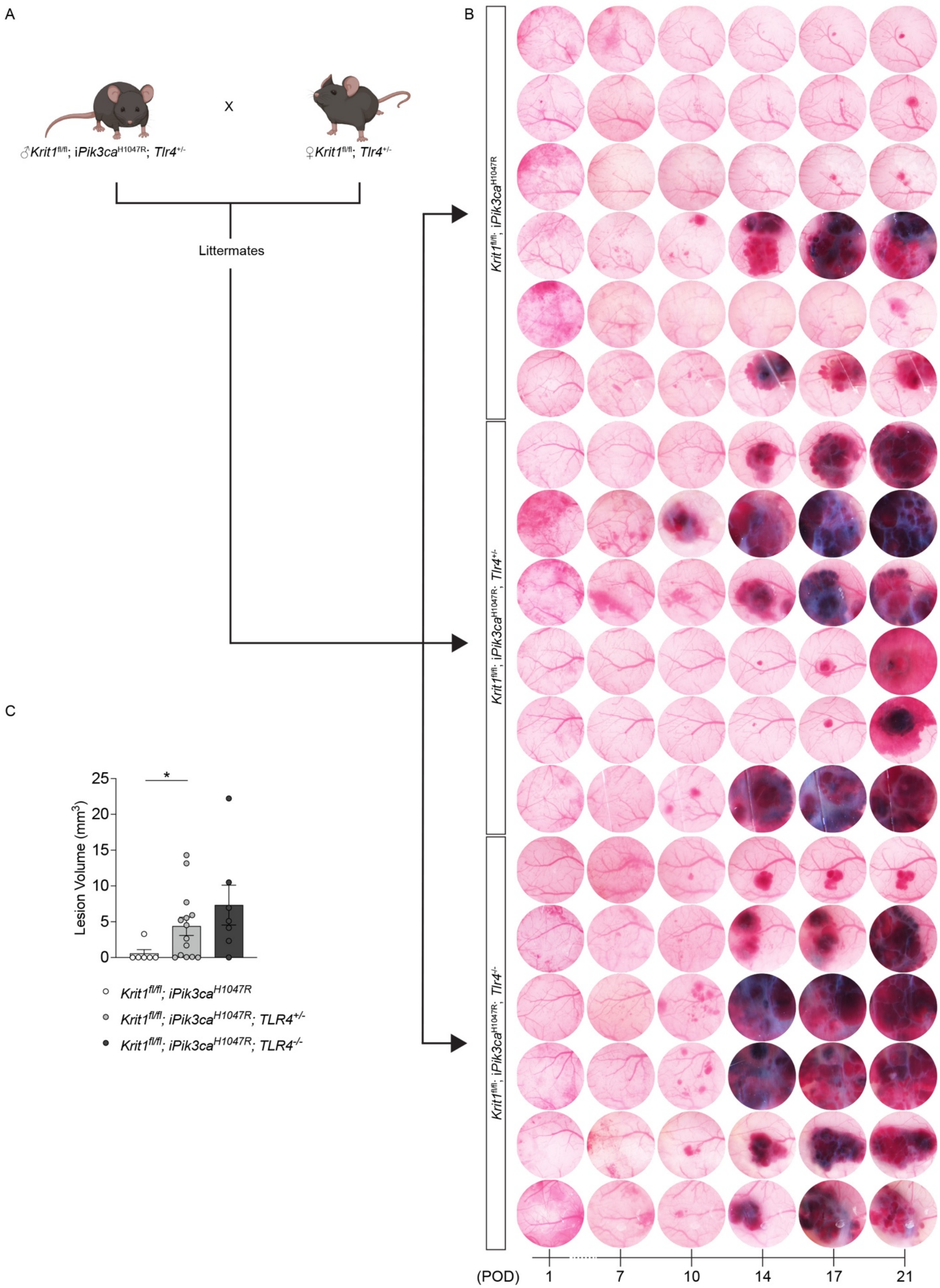
A
X
♂Krit1fl/fl; iPik3caH1047R; Tlr4+/-
♀Krit1fl/fl; Tlr4+/-
Littermates
B
Krit1fl/fl; iPik3caH1047R
Krit1fl/fl; iPik3caH1047R; Tlr4+/-
Krit1fl/fl; iPik3caH1047R; Tlr4-/-
(POD) 1 7 10 14 17 21
C
Lesion Volume (mm3)
25
20
15
10
5
0
*
Krit1fl/fl; iPik3caH1047R
Krit1fl/fl; iPik3caH1047R; TLR4+/-
Krit1fl/fl; iPik3caH1047R; TLR4-/-

Supplemental Figure 2. Live-animal imaging of CCM lesion formation in mice with global TLR4 deficient background. (A) Schematic representation of the breeding strategy used to generate littermate cohorts of the indicated genotypes for experimental analysis. (B) Representative serial brightfield images obtained through cranial windows demonstrating CCM lesion growth in *Krit1*$^{fl/fl}$; i*Pik3ca*^{H1047R}, *Krit1*$^{fl/fl}$; i*Pik3ca*^{H1047R}; *Tlr4*$^{+/-}$ and *Krit1*$^{fl/fl}$; i*Pik3ca*^{H1047R}; *Tlr4*$^{-/-}$ mice from POD 1 to POD 21 following focal AAV-Cre delivery. Images illustrate longitudinal *in vivo* monitoring of lesion initiation and progression. (C) MicroCT quantification of CCM lesion volumes in *Krit1*$^{fl/fl}$; i*Pik3ca*^{H1047R} (n=6), *Krit1*$^{fl/fl}$; i*Pik3ca*^{H1047R}; *Tlr4*$^{+/-}$ (n=14), and *Krit1*$^{fl/fl}$; i*Pik3ca*^{H1047R}; *Tlr4*$^{-/-}$ (n=7) mouse brains harvested on POD 21. Data shown are means ± SEM. *$p < 0.05$ by Welch ANOVA, followed by Dunnett's T3 *post-hoc* multiple comparison test.
